# Comparison and evaluation of percutaneous transforaminal endoscopic discectomy treatment efficacy in patients with lumbar disc herniation of different age groups

**DOI:** 10.3389/fsurg.2025.1587857

**Published:** 2025-05-15

**Authors:** Xiulei Xu, Jie Song, Gang Zhou, Jun Li, Xiaorui Zhang

**Affiliations:** Department of Orthopedic, Zhejiang University, Sir Run Run Shaw, Alar Hospital of Xinjiang Production and Construction Corps, Alar City, Xinjiang, China

**Keywords:** lumbar disc herniation (LDH), percutaneous transforaminal endoscopic discectomy (PTED), age, generalized linear mixed model (GLMM), clinical outcome

## Abstract

**Background:**

Lumbar disc herniation (LDH) is also a degenerative disease of the spine, and age is an important factor affecting the prognosis of LDH patients. The aim of this study is to analyze the pain levels and physical function changes of LDH patients in different age groups before and after percutaneous transforaminal endoscopic discectomy (PTED) treatment, and to analyze the factors that affect postoperative clinical outcome indicators.

**Method:**

This study included 100 LDH patients. Collect baseline characteristics of patients and compare the differences in pain levels, ODI scores, JOA scores, BBS scores, and SF-36 scores between LDH patients of different ages before and after PTED treatment. The generalized linear mixed model (GLMM) was used to analyze the impact of different factors on postoperative clinical outcomes, and the ROC curve was used to evaluate the predictive ability of age on postoperative indicator improvement.

**Result:**

The research results indicate that after PTED treatment, the postoperative clinical outcomes of all patients have significantly improved, with the younger group showing the most significant improvement. In the young group, the amount of intraoperative bleeding, the length of operation, the length of postoperative bed rest, and the incidence rate of postoperative complications were the lowest. GLMM analysis showed that follow-up time, Pfirrmann grading, baseline NLR level, age, lumbar spondylolisthesis, affected intervertebral disc segment L5/S1, and the interaction between age and follow-up time were significant influencing factors for postoperative clinical outcomes. As the follow-up time prolongs, the influence of age on ODI and JOA gradually weakens. ROC curve analysis showed that age had the strongest predictive ability for the improvement of preoperative and postoperative ODI scores and JOA scores, with AUC values of 0.641 and 0.646, respectively.

**Conclusion:**

The clinical outcomes of PTED treatment in young patients showed the most significant improvement. With the extension of postoperative follow-up time, the influence of age on postoperative clinical outcomes gradually decreases. This study also provides valuable reference for exploring the factors that affect the therapeutic effect of PTED.

## 1 Introduction

Lumbar Disc Herniation (LDH) is a common degenerative disease of the spine, especially in the middle-aged and elderly people with a high incidence rate (1, 2). With the acceleration of global aging process, the incidence rate of LDH is increasing year by year, and seriously affects the quality of life of patients (3). The clinical manifestations of lumbar disc herniation include lower back pain, sciatica, lower limb numbness, decreased muscle strength, etc. In severe cases, it can lead to limited motor function, affecting the patient's daily life and work ability (4). In clinical practice, the treatment methods for lumbar disc herniation (LDH) can be divided into conservative treatment and surgical treatment (5). Conservative treatment includes drug therapy (such as nonsteroidal anti-inflammatory drugs, muscle relaxants), physical therapy (such as traction, rehabilitation exercises) (6), nerve block, lifestyle adjustments, etc. (7), usually used for patients with mild or moderate symptoms. For patients with severe symptoms, ineffective conservative treatment, or nerve damage, surgical treatment becomes a necessary choice. Common surgical methods include traditional discectomy, minimally invasive surgery (such as PTED), as well as disc replacement surgery, spinal fusion surgery, etc. (8, 9).

With the continuous advancement of treatment technology, PTED (percutaneous transforaminal endoscopic discectomy) has gradually become a commonly used treatment option for LDH patients as a minimally invasive surgical method (10). Microdiscectomy, also a minimally invasive surgery, requires cutting through the skin, muscles, and some ligaments. It is suitable for patients with a larger disc herniation or more severe disc degeneration. Although the surgical trauma is smaller than traditional open surgery, it still requires a relatively larger incision and exposure of surrounding tissues, resulting in a longer recovery time. PTED has the advantages of less trauma, faster recovery, shorter hospital stay, and fewer complications, making it one of the preferred surgeries for treating lumbar disc herniation (11, 12). Through PTED, doctors can enter the intervertebral disc area through small incisions and use endoscopic techniques to remove protruding intervertebral disc tissue, reducing pressure on nerve roots or spinal cord, effectively relieving pain and improving function.

The efficacy of PTED is influenced by various factors, such as age, history of diabetes, duration of symptoms, smoking, and alcohol consumption (13). Case-related factors, such as the location, size, and type of disc herniation, as well as the degree of disc degeneration, also significantly affect the outcomes of PTED (14, 15). Age is an important factor affecting the prognosis of LDH patients, as elderly patients may face longer recovery periods, higher risk of complications, and poorer postoperative outcomes after surgery. Therefore, understanding the impact of age on the treatment efficacy of PTED and evaluating the postoperative clinical outcomes of patients in different age groups has important clinical significance. In addition, we also conducted further analysis of the interaction between age and follow-up time to more specifically illustrate which time period age has the most significant impact on clinical outcomes.

## 2 Materials and methods

### 2.1 Patient selection

This study is a retrospective study that included patients with lumbar disc herniation (LDH) who underwent percutaneous transforaminal endoscopic discectomy (PTED) treatment at our hospital from January 2019 to December 2023. All patients were diagnosed with lumbar disc herniation through imaging examinations (MRI or CT), and the symptoms persisted for more than 6 weeks. The inclusion criteria are: Single-level Lumbar Disc Herniation; the affected segment is L3/L4, L4/L5, L5/S1; Pfirrmann classification is level three or above; Conservative treatment is ineffective. The exclusion criteria are osteoporosis and other bone diseases that affect surgical safety and efficacy; Severe spinal deformities, spinal tumors, infections or surgical contraindications, as well as accompanying serious systemic diseases (such as cancer, heart failure, etc.); Accompanied by severe systemic diseases such as end-stage cancer, severe cardiovascular disease, liver and kidney failure, etc. Patients were divided into three groups based on age: the young group (20–40 years), the middle-aged group (40–60 years), and the elderly group (over 60 years).

### 2.2 Treatment methods

After general anesthesia, the patient is placed in a prone position. The culprit disc space is located using x-ray (G-arm) guidance. A needle is inserted at predetermined marked points, and the position is adjusted under x-ray guidance to ensure accurate placement. The dilator is gradually inserted through the guidewire, followed by a 5 mm incision. The trephine sheath and medium-sized trephine are then inserted to enlarge the intervertebral foramen. Under transforaminal endoscope observation, the surgeon can accurately locate the herniated disc under real-time visualization, examine the site of disc herniation, and use specialized surgical instruments to remove the herniated nucleus pulposus and perform annuloplasty. Finally, withdraw the transforaminal endoscopic system, remove the working cannula, and close the incision with sutures. After the surgery, the patient is observed in the hospital for several hours to one night. During the postoperative recovery phase, regular rehabilitation exercises and follow-up assessments are performed.

### 2.3 Data collection

Baseline characteristics of patients were collected, including age, gender, body mass index (BMI), basic diseases (such as diabetes, hypertension, cardiovascular disease), smoking and drinking history, calcification of intervertebral disc, involved intervertebral disc segment and Pfirmann classification. The clinical outcome indicators before surgery, 1 month after surgery, 3 months after surgery, and 6 months after surgery include pain level (VAS visualization score), functional impairment [Oswestry Disability Index, ODI score, Japanese Orthopaedic Association, JOA score (16)], balance function [Berg Balance Scale, BBS score (17)], and quality of life (SF-36 score), surgical time, intraoperative blood loss, postoperative bed rest time, and postoperative complications (such as infection, nerve injury, etc.). We define a decrease of 3 points or more in VAS score before and 6 months after surgery as an improvement in pain level, and a decrease of 40% or more in ODI score as an improvement in ODI score. An increase of 8 points or more in JOA score is considered an improvement in JOA score. An increase of 5 or more points in BBS rating is considered an improvement in BBS rating. An increase of more than 20 points in SF-36 score is considered an improvement in SF-36 score.

### 2.4 Statistical analysis

Use R 4.4.0 statistical software for data analysis. Count data is expressed in frequency (percentage), and comparison between groups is performed using chi square test. Continuous variables are represented by median (minimum-maximum), and inter group comparisons are performed using one-way analysis of variance (ANOVA) or Kruskal Wallis H test. For the analysis of influencing factors on postoperative clinical outcomes, a generalized linear mixed model (GLMM) was used for regression analysis to control for potential confounding factors. Evaluate the predictive role of age on PTED outcomes using receiver operating characteristic (ROC) curve.

## 3 Results

### 3.1 Baseline characteristics of LDH patients

This study included 100 patients with lumbar disc herniation (LDH), with male patients accounting for 53% and female patients accounting for 47%. The median body mass index (BMI) of the patient is 25.0, and the median duration of the disease is 4.0 years. Diabetes accounted for 13%, hypertension 9% and cardiovascular disease 19%. Moderate smoking and alcohol consumption accounted for 51% and 52% of patients, respectively, while severe smoking and alcohol consumption accounted for 36% and 33%, respectively. 6% of patients have intervertebral disc calcification. The main affected segments of the patient were L4/L5 (61%), followed by L5/S1 (29%) and L3/L4 (10%). Among patients with lumbar spondylolisthesis, 86% did not experience spondylolisthesis, and 11% had grade I spondylolisthesis. In the Pfirrmann grading system, 75% of patients are classified as grade III and 23% as grade IV. The median white blood cell count was 6.9, the median NLR was 1.9, the median ESR was 10.7, and the LDL and HDL were 2.2 and 1.4, respectively (Table 1).

**Table 1:** Baseline information of LDH patients of different age groups.

Variables	All Patients (*n* = 100)	Young (*n* = 20)	Middle-aged (*n* = 48)	Older (*n* = 32)	*P*-value
Gender					0.327691
Male	53 (53%)	10 (50%)	29 (60.42%)	14 (43.75%)	
Female	47 (47%)	10 (50%)	19 (39.58%)	18 (56.25%)	
BMI	25.0 (19.6–30.1)	24.3 (19.6–30.0)	25.1 (19.6–30.1)	25.6 (19.6–30.1)	0.399
Duration of disease (year)	4.0 (0.6–8.0)	3.2 (0.6–8.0)	3.9 (0.6–8.0)	4.6 (0.6–7.9)	0.315
Diabetes					0.190904
Yes	13 (13%)	2 (10%)	4 (8.33%)	7 (21.88%)	
No	87 (87%)	18 (90%)	44 (91.67%)	25 (78.12%)	
Hypertension					0.495556
Yes	9 (9%)	1 (5%)	6 (12.5%)	2 (6.25%)	
No	91 (91%)	19 (95%)	42 (87.5%)	30 (93.75%)	
Cardiovascular diseases					0.202157
Yes	19 (19%)	1 (5%)	11 (22.92%)	7 (21.88%)	
No	81 (81%)	19 (95%)	37 (77.08%)	25 (78.12%)	
Smoking					0.444808
Mild	13 (13%)	2 (10%)	9 (18.75%)	2 (6.25%)	
Moderate	51 (51%)	12 (60%)	23 (47.92%)	16 (50%)	
Severe	36 (36%)	6 (30%)	16 (33.33%)	14 (43.75%)	
Alcohol consumption					0.312722
Mild	15 (15%)	1 (5%)	8 (16.67%)	6 (18.75%)	
Moderate	52 (52%)	14 (70%)	25 (52.08%)	13 (40.62%)	
Severe	33 (33%)	5 (25%)	15 (31.25%)	13 (40.62%)	
Calcification of the herniated disc					0.616378
Yes	6 (6%)	1 (5%)	2 (4.17%)	3 (9.38%)	
No	94 (94%)	19 (95%)	46 (95.83%)	29 (90.62%)	
Affected segment					0.391475
L3/L4	10 (10%)	4 (20%)	3 (6.25%)	3 (9.38%)	
L4/L5	61 (61%)	9 (45%)	32 (66.67%)	20 (62.5%)	
L5/S1	29 (29%)	7 (35%)	13 (27.08%)	9 (28.12%)	
Lumbar spondylolisthesis					0.113756
No	86 (86%)	17 (85%)	44 (91.67%)	25 (78.12%)	
Grade I	11 (11%)	3 (15%)	4 (8.33%)	4 (12.5%)	
Grade II	3 (3%)	0 (0%)	0 (0%)	3 (9.38%)	
Pfirrmann grading					0.731816
Grade III	75 (75%)	15 (75%)	34 (70.83%)	26 (81.25%)	
Grade IV	23 (23%)	5 (25%)	13 (27.08%)	5 (15.62%)	
Grade V	2 (2%)	0 (0%)	1 (2.08%)	1 (3.12%)	
WBC (10^9^/L)	6.9 (4.8–8.6)	7.1 (4.9–8.5)	6.8 (4.8–8.6)	6.7 (4.8–8.6)	0.33
NLR (mg/L)	1.9 (1.2–2.7)	1.8 (1.2–2.7)	2.0 (1.2–2.7)	2.0 (1.2–2.7)	0.276
ESR (mm/h)	10.7 (2.7–17.1)	10.2 (2.9–16.9)	10.1 (2.7–17.0)	11.4 (2.8–17.1)	0.154
LDL (mmol/L)	2.2 (1.4–3.1)	2.2 (1.4–3.1)	2.2 (1.4–3.1)	2.2 (1.4–3.1)	0.948
HDL (mmol/L)	1.4 (1.1–1.7)	1.4 (1.1–1.7)	1.4 (1.1–1.7)	1.4 (1.1–1.7)	0.833

BMI, body mass index; WBC, white blood cell count; NLR, neutrophil-to-lymphocyte ratio; ESR, erythrocyte sedimentation rate; LDL, low-density lipoprotein; HDL, high-density lipoprotein.

### 3.2 Differences in pain and functional impairment before and after treatment in LDH patients of different age groups

In terms of pain levels, all patients showed significant pain relief at T1, T2, and T3 compared to baseline (T0), with the younger group showing the most significant improvement. For the Oswestry Disability Index (ODI), all groups showed a significant reduction in functional impairment between T0 and T3, with the elderly group showing the greatest decrease at T1 and T2. The JOA score indicates a significant improvement in all age groups from T0 to T3, with larger improvements observed in the younger and middle-aged groups. The BBS scores of all groups also significantly increased, indicating a significant improvement in balance function, with the young and middle-aged groups showing greater improvement than the elderly group. The SF-36 score (quality of life) improved in all groups, with the younger group showing the most significant improvement. Overall, the data shows that pain, functional impairment, balance ability, and quality of life have significantly improved in all age groups, with the younger group showing the best improvement effect (Table 2).

**Table 2:** The differences in pain and functional disability before and after treatment in LDH patients of different age groups.

Indicators	All patients (*n* = 100)	Young (*n* = 20)	Middle-aged (*n* = 48)	Older (*n* = 32)	*P*-value
VAS					
T0	7 (5–9)	7 (5–9)	7 (5–9)	7 (5–9)	0.563
T1	3 (1–4)	2 (1–4)	3 (1–4)	3 (1–4)	0.00846
T2	2 (0–4)	1 (0–4)	2 (0–4)	2 (0–4)	0.00745
T3	2 (0–3)	1 (0–3)	2 (0–3)	2 (0–3)	0.0166
Oswestry Disability Index, ODI					
T0	48 (38–57)	47 (38–56)	48 (38–57)	49 (38–57)	0.565
T1	28 (18–37)	27 (19–37)	27 (18–37)	31 (18–37)	0.00172
T2	20 (11–28)	19 (11–28)	19 (11–28)	21 (12–28)	0.0331
T3	16 (9–23)	15 (9–23)	16 (9–23)	17 (9–23)	0.0351
Japanese Orthopaedic Association, JOA					
T0	11 (8–14)	11 (8–14)	11 (8–14)	11 (8–14)	0.53
T1	22 (18–25)	22 (18–25)	22 (18–25)	21 (18–25)	0.0209
T2	24 (20–28)	25 (21–28)	24 (20–28)	23 (20–28)	0.00136
T3	24 (20–28)	25 (20–28)	24 (20–28)	24 (20–28)	0.0488
Berg Balance Scale (BBS)					
T0	29 (21–37)	30 (21–37)	30 (21–37)	29 (21–37)	0.687
T1	41 (33–48)	42 (33–48)	41 (33–48)	40 (33–48)	0.0106
T2	43 (36–50)	43 (36–50)	43 (36–50)	42 (36–50)	0.0354
T3	46 (39–54)	47 (39–54)	46 (39–54)	44 (39–54)	0.0351
SF-36					
T0	50 (43–57)	50 (43–57)	50 (43–57)	50 (43–57)	0.947
T1	63 (56–71)	65 (56–71)	63 (56–71)	62 (56–71)	0.0294
T2	76 (69–82)	78 (69–82)	77 (69–82)	75 (69–82)	0.0223
T3	82 (74–90)	84 (74–90)	82 (74–90)	82 (74–90)	0.0183

T0, Before surgery; T1, 1 month after surgery; T2, 3 months after surgery; T3, 6 months after surgery.

### 3.3 Differences in perioperative indicators and postoperative complications among LDH patients of different age groups

The results showed significant differences in intraoperative bleeding, surgical time, and postoperative bed rest time among different age groups, with elderly patients having significantly higher bleeding, surgical time, and bed rest time than the other two groups. In terms of postoperative complications, the elderly group had the highest incidence of infection, and there was a significant difference compared to the other two groups (*P* = 0.0374). Nerve injury occurred in one elderly patient, with no significant difference (*P* = 0.34). The incidence of recurrent lumbar disc herniation and spinal instability is also significantly higher in the elderly group (*P* = 0.00649, *P* = 0.01195) (Table 3).

**Table 3:** The differences in perioperative indicators and postoperative complications among LDH patients of different age groups.

Surgical outcomes	All patients (*n* = 100)	Young (*n* = 20)	Middle-aged (*n* = 48)	Older (*n* = 32)	*P*-value
Intraoperative blood loss (ml)	34.4 (21.1–48.9)	29.1 (21.1–48.8)	33.9 (21.2–48.9)	37.0 (21.1–48.2)	0.00053
Operative time (min)	55 (33–75)	50 (33–72)	55 (33–75)	59 (33–75)	0.00023
Postoperative bed rest time (h)	9 (5–13)	8 (5–13)	8 (5–13)	10 (5–13)	5.52 × 10^−5^
Infection					0.0374
Yes	3 (3%)	0 (0%)	0 (0%)	3 (9.375%)	
No	97 (97%)	20 (100%)	48 (100%)	29 (90.625%)	
Nerve injury					0.34
Yes	1 (1%)	0 (0%)	0 (0%)	1 (3.125%)	
No	99 (99%)	20 (100%)	48 (100%)	31 (96.875%)	
Recurrent disc herniation within 1 year					0.00649
Yes	7 (7%)	0 (0%)	1 (2.08%)	6 (18.75%)	
No	93 (93%)	20 (100%)	47 (97.92%)	26 (81.25%)	
Spinal instability					0.01195
Yes	4 (4%)	0 (0%)	0 (0%)	4 (12.5%)	
No	96 (96%)	20 (100%)	48 (100%)	28 (87.5%)	

### 3.4 GLMM analysis of factors affecting postoperative clinical outcome indicators

For postoperative pain, follow-up time (T1, T2, T3), Pfirrmann grading, baseline NLR level, and age are significant factors, indicating that the higher the levels of these factors, the greater the degree of postoperative pain. The *p*-values of other factors such as intervertebral disc calcification (*p* = 0.437), affected intervertebral disc segments (L4/L5, L5/S1), lumbar spondylolisthesis, etc. are all greater than 0.05, indicating that these variables are not significantly related to postoperative pain. For postoperative functional impairment, the affected intervertebral disc segment with L5/S1 will significantly increase the ODI score, and the older the age, the higher the postoperative functional impairment score. Compared to preoperative follow-up, the longer the postoperative follow-up time, the greater the impact on ODI score, and the interaction between age and follow-up time is significant. The B value decreases with time, indicating that age has a decreasing effect on ODI score over time. For the JOA score, Pfirrmann grading, follow-up time (T1, T2, T3), and age have a significant impact. The higher the Pfirrmann rating, the lower the JOA score. The impact of time effect on JOA is gradually increasing. The interaction between age and follow-up time indicates that the interaction with age is most significant at T1. For BBS, patients with lumbar spondylolisthesis have lower BBS scores, and the older they are, the lower their BBS scores. The effect of time on BBS gradually increases, and the interaction between age and time is not significant. For SF-36 scores, the higher the age, the lower the score, and the time effect gradually increases. The interaction between age and T1 is close to a significant level, indicating that age has a significant impact on quality of life at T1 (Table 4).

**Table 4:** GLMM analysis of factors affecting postoperative clinical outcome indicators.

Outcome measures	Model outputs	Calcification of the herniated disc	Affected segmentL4/L5	Affected segmentL5/S1	Lumbar spondylolisthesis	Pfirrmann grading	WBC	NLR	ESR	LDL	HDL	Age	TimeT1	TimeT2	TimeT3	Age* TimeT1	Age* TimeT2	Age* TimeT3
VAS	Estimate	−0.048	−0.062	−0.064	−0.016	0.119	0.013	0.092	0.003	0.012	−0.029	0.214	−1.079	−1.445	−1.603	0.042	0.113	0.075
	Std error	0.062	0.049	0.052	0.035	0.030	0.015	0.036	0.004	0.034	0.093	0.044	0.083	0.084	0.082	0.062	0.063	0.062
	Statistic	−0.777	−1.263	−1.223	−0.444	3.967	0.866	2.556	0.807	0.366	−0.309	4.864	−13.049	−17.260	−19.456	0.677	1.791	1.216
	*P* value	0.437	0.207	0.221	0.657	0.000	0.387	0.011	0.419	0.715	0.758	0.000	0.000	0.000	0.000	0.498	0.073	0.224
ODI	Estimate	0.029	0.034	0.054	−0.013	0.020	0.008	−0.001	0.001	0.015	0.027	0.040	−0.612	−0.965	−1.146	0.057	0.056	0.054
	Std error	0.023	0.018	0.019	0.013	0.011	0.005	0.013	0.001	0.012	0.035	0.016	0.029	0.029	0.029	0.022	0.022	0.022
	Statistic	1.288	1.883	2.842	−0.963	1.819	1.400	−0.070	0.510	1.194	0.795	2.500	−20.765	−32.759	−39.022	2.597	2.498	2.419
	*P* value	0.198	0.060	0.004	0.336	0.069	0.162	0.944	0.610	0.232	0.427	0.012	0.000	0.000	0.000	0.009	0.012	0.016
JOA	Estimate	0.010	0.004	−0.002	−0.004	−0.012	0.004	−0.008	0.000	0.004	−0.010	−0.026	0.722	0.845	0.837	−0.051	−0.046	−0.039
	Std error	0.012	0.009	0.010	0.007	0.006	0.003	0.007	0.001	0.006	0.018	0.009	0.016	0.016	0.016	0.012	0.012	0.012
	Statistic	0.873	0.430	−0.228	−0.645	−2.178	1.279	−1.142	0.222	0.562	−0.533	−2.889	45.546	53.413	52.852	−4.304	−3.883	−3.227
	*P* value	0.383	0.667	0.820	0.519	0.029	0.201	0.254	0.825	0.574	0.594	0.004	0.000	0.000	0.000	0.000	0.000	0.001
BBS	Estimate	−0.004	−0.003	−0.002	−0.015	0.002	0.001	0.001	0.000	0.005	−0.029	−0.026	0.323	0.389	0.457	−0.001	0.000	0.004
	Std error	0.012	0.010	0.010	0.007	0.006	0.003	0.007	0.001	0.007	0.018	0.009	0.016	0.016	0.016	0.012	0.012	0.012
	Statistic	−0.355	−0.301	−0.182	−2.183	0.273	0.406	0.188	−0.028	0.726	−1.551	−2.939	19.739	23.785	27.953	−0.069	0.009	0.352
	*P* value	0.723	0.764	0.856	0.029	0.785	0.685	0.851	0.977	0.468	0.121	0.003	0.000	0.000	0.000	0.945	0.993	0.725
SF-36	Estimate	0.001	−0.002	−0.007	0.002	0.003	0.000	−0.009	0.001	0.001	0.015	−0.013	0.255	0.429	0.513	−0.013	−0.008	−0.012
	Std error	0.007	0.006	0.006	0.004	0.003	0.002	0.004	0.000	0.004	0.011	0.005	0.009	0.009	0.009	0.007	0.007	0.007
	Statistic	0.213	−0.300	−1.219	0.526	0.791	−0.153	−2.164	1.805	0.329	1.358	−2.600	28.661	48.292	57.642	−1.944	−1.242	−1.745
	*P* value	0.831	0.764	0.223	0.599	0.429	0.878	0.030	0.071	0.742	0.174	0.009	0.000	0.000	0.000	0.052	0.214	0.081

WBC, white blood vell count; NLR, neutrophil-to-lymphocyte ratio; ESR, erythrocyte sedimentation rate; LDL, low-density lipoprotein; HDL, high-density lipoprotein; T0, Before surgery; T1, 1 month after surgery; T2, 3 months after surgery; T3, 6 months after surgery; VAS, pain level visual analogue scale; ODI, oswestry disability index; JOA, Japanese orthopaedic association; BBS, berg balance scale; SF-36, short form 36.

### 3.5 ROC curve analysis of the predictive ability of age for improvement in postoperative clinical outcome indicators

**Figure 1:**
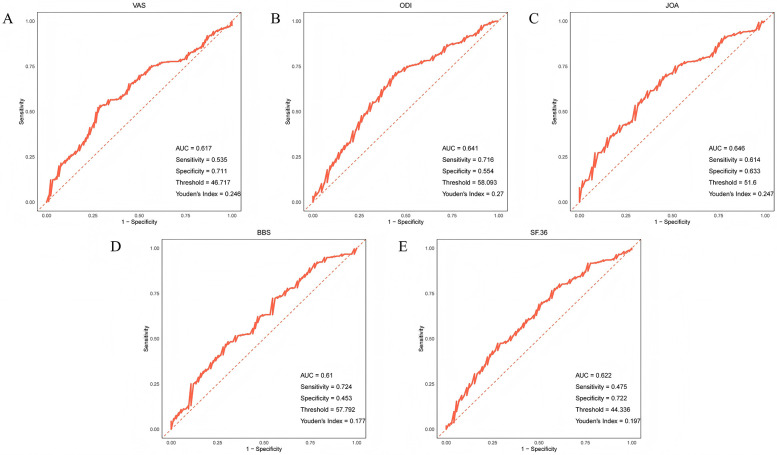
ROC curve of age prediction **(A)** pain level **(B)** ODI score **(C)** JOA score **(D)** BBS score **(E)** SF-36 score improvement.

The results showed that age had strong predictive performance for the improvement of preoperative and postoperative pain levels, preoperative and postoperative ODI scores, preoperative and postoperative JOA scores, preoperative and postoperative BBS scores, and preoperative and postoperative SF-36 scores. Among them, age had the strongest predictive ability for the improvement of ODI scores and JOA scores, with AUC values of 0.641 and 0.646, respectively (Figures 1A–E).

## 4 Discussion

Our research found that after PTED treatment, patients' pain levels and functional impairments were significantly improved. This is because PTED is a minimally invasive surgery that uses small incisions and endoscopic techniques to remove intervertebral discs, avoiding the extensive trauma of traditional open surgery. This minimally invasive nature not only reduces intraoperative injuries, but also significantly reduces the risk of postoperative complications such as infection and bleeding, which helps patients recover function more quickly. This helps patients recover lumbar spine function early, reduce postoperative pain, and improve quality of life. In addition, age has the strongest predictive power for the improvement of ODI and JOA indicators after PTED treatment, which also indicates that age plays an important role in the changes of these two scores. This may be because young patients have higher tissue elasticity and metabolism, stronger immune system, and fewer chronic diseases (such as diabetes, hypertension, etc.) that affect postoperative recovery (18).

The GLMM results showed that follow-up time, Pfirrmann grading, baseline NLR (neutrophil to lymphocyte ratio) level, age, lumbar spondylolisthesis, affected intervertebral disc segment L5/S1, and the interaction between age and follow-up time were significant influencing factors for postoperative clinical outcomes. Patients with severe disc degeneration (Pfirrmann grade higher) may experience more severe nerve compression or disc degeneration, leading to slower postoperative recovery and lower degree of functional improvement (19). Patients with higher baseline NLR levels also experience higher levels of postoperative pain, which may be due to a stronger systemic inflammatory response in their bodies (20). This inflammatory response may affect the function of the nervous system, slow down tissue repair, increase postoperative pain perception, and prolong the duration of pain. Patients with lumbar spondylolisthesis have lower postoperative BBS scores. Lumbar spondylolisthesis refers to the forward or backward displacement of one vertebral body relative to another, which can affect the stability of the spine (21). Although PTED treatment can alleviate nerve compression and pain, patients may still experience certain nerve root compression and spinal instability, which can lead to sensory or motor impairments in the lower limbs after surgery, resulting in decreased balance function. The affected segment is L5/S1, which has a higher postoperative ODI score compared to other parts. This may be because the L5/S1 intervertebral disc segment is the most common site of lumbar disc herniation and is most susceptible to compression (22). The L5/S1 segment is closely related to lower limb movement and sensation, with the L5 nerve root innervating the extensor muscles of the dorsum of the foot and some ankle functions (23), while the S1 nerve root innervating the flexor muscles of the sole, ankle, and calf. The protrusion of L5/S1 can cause nerve compression in these areas, and postoperative recovery may take a long time.

One of the highlights of our research is the analysis of the interaction between age and follow-up time. The results show that the influence of age on postoperative ODI score and JOA score decreases over time. This finding suggests that the impact of age on postoperative function may be dynamically changing after PTED treatment. As the follow-up time prolongs, postoperative recovery becomes less affected by age. In the early stages of surgery, elderly patients may experience more significant functional impairment and pain. Over time, the recovery of elderly patients, like young patients, tends to stabilize. This interaction analysis has important clinical practical significance, which indicates that long-term follow-up of elderly patients is particularly important. Doctors can adjust rehabilitation strategies based on the performance during follow-up to help patients better adapt to postoperative life and improve their quality of life.

This study holds great significance. As a minimally invasive technique, PTED has been receiving increasing attention; however, current research on this new approach remains limited. Our study explores the impact of age on the effectiveness of PTED, which may help with preoperative patient selection and prognosis assessment. It also provides a basis for developing individualized treatment strategies for patients of different age groups. The findings lay a foundation for further validation of the potential advantages of PTED and its broader application in diverse patient populations within the field of neurosurgery. This study also has certain limitations. Firstly, due to its retrospective nature, there is a certain degree of data selection bias. Secondly, the research scale is limited and there has been no in-depth study on the mechanism of the interaction between age and follow-up time. Therefore, larger scale randomized controlled trials can be conducted in the future to validate the conclusions of this study.

## 5 Conclusion

This study retrospectively included 100 patients with LDH and divided them into young patients, middle-aged patients, and elderly patients according to their age. After PTED treatment, all patients showed significant improvement in clinical outcomes, with the best improvement observed in young patients. Other factors such as Pfirrmann grading and baseline NLR levels can also affect the clinical outcomes of LDH patients. With the extension of postoperative follow-up time, the influence of age on postoperative clinical outcomes gradually decreases. These findings provide scientific evidence for the development of personalized treatment plans for LDH patients in the future. However, despite the significant clinical application value of these results, due to the limitations of the retrospective study, further validation of these results is needed in future prospective studies.

## Data Availability

The original contributions presented in the study are included in the article/Supplementary Material, further inquiries can be directed to the corresponding author.
